# Cancer Vaccines in the World of Immune Suppressive Monocytes (CD14^+^HLA-DR^lo/neg^ Cells): The Gateway to Improved Responses

**DOI:** 10.3389/fimmu.2014.00147

**Published:** 2014-04-04

**Authors:** Rebecca R. Laborde, Yi Lin, Michael P. Gustafson, Peggy A. Bulur, Allan B. Dietz

**Affiliations:** ^1^Human Cellular Therapy Laboratory, Division of Transfusion Medicine, Department of Laboratory Medicine and Pathology, Mayo Clinic, Rochester, MN, USA; ^2^Division of Hematology, Department of Medicine, Mayo Clinic, Rochester, MN, USA

**Keywords:** CD14^+^HLA-DR^lo/neg^, MDSC, dendritic cells, immunotherapy, monocytes

## Abstract

Dendritic cells are an important target in cancer immunotherapy based on their critical role in antigen presentation and response to tumor development. The capacity of dendritic cells to stimulate anti-tumor immunity has led investigators to use these cells to mediate anti-tumor responses in a number of clinical trials. However, these trials have had mixed results. The typical method for generation of *ex vivo* dendritic cells starts with the purification of CD14^+^ cells. Our studies identified a deficiency in the ability to generate mature dendritic cell using CD14^+^ cells from cancer patients that corresponded with an increased population of monocytes with altered surface marker expression (CD14^+^HLA-DR^lo/neg^). Further studies identified systemic immune suppression and increased concentrations of CD14^+^HLA-DR^lo/neg^ monocytes capable of inhibiting T-cell proliferation and DC maturation. Together, these findings strongly suggest that protocols aimed at immune stimulation via monocytes/dendritic cells, if optimized on normal monocytes or in systems without these suppressive monocytes, are unlikely to engender effective DC maturation *in vitro* or efficiently trigger DC maturation *in vivo*. This highlights the importance of developing optimal protocols for stimulating DCs in the context of significantly altered monocyte phenotypes often seen in cancer patients.

## Dendritic Cells as Cancer Vaccines

Dendritic cells are potent signal transducers in the immune system. These cells present antigen, are the essential bridge between the innate and adaptive arms of the immune system, and serve as regulators to modulate immune response to pathogenic invasion, tissue injury, and tumor development. As such, dendritic cells have received significant focus as a promising vehicle for the development of vaccines for cancer immunotherapy. We now have a more complete understanding of DC ontology with the realization that DCs exist in diverse subsets, all capable of activating T cells but possessing unique functions. DCs are classified into two broad categories. The first are monocyte-derived DCs resulting from stimulation due to inflammation or infection. The second category are steady-state DCs which include resident CD8^+^ DCs located in the thymus, resident CD8^−^ DCs in the spleen, plasmacytoid DCs (pDCs), migratory DCs, and Langerhans cells [reviewed in Ref. (1, 2)]. Each of these classes of DCs has been demonstrated to play a key role in immune surveillance and response but for the purpose of DC-based vaccines for immunotherapy in cancer, the focus has been on CD14^+^ monocyte-derived DCs.

The *in vivo* pathways associated with the development of dendritic cells from monocyte precursors and the mechanisms and consequences of pathogenic activation have been described (3, 4). Briefly, DCs arise from monocyte progenitors into an immature state (iDC) responsible for immune surveillance via pathogen detection. Once activated, iDC further differentiate into mature dendritic cells (mDC) and travel to lymph nodes to activate the adaptive (typically T- and B-cell responses) and innate immune response (5). DCs also play a role in limiting the immune response against self antigen (self-tolerance) as well as limiting response to tissue damage in the absence of pathogenic signals (6). iDC can suppress immunity and have been shown to be capable of eliminating antigen-specific T cells (7). Restriction of the capacity of iDC to differentiate into mDC has been a mechanism used by viruses, parasites, and bacteria to maintain a state of self-tolerance and to enable microbial pathology (8–10). Thus, manipulation and maintenance of a state of iDC with a block on the ability to differentiate into mDC is a key mechanism of immune suppression.

To generate mDC *in vitro* for clinical use, the CD14^+^ monocytes are the preferred precursor due to their abundance and ease of collection. CD14^+^ monocytes are purified from mononuclear cells via adherence to plastic, antibody selection, or size centrifugation and used as source material to differentiate DC. To drive the immune response, the DCs are pulsed with tumor antigens in the form of peptides, RNA, or lysates derived from whole tumors or cell lines (11, 12). Additionally, viruses can be a potent mechanism to deliver tumor antigens (13–15). Manufacturing methods reported among clinical trials vary greatly. As a variety of methods with subtle optimizations of DC cultures have been published, there are few constants (DC activation state, tumor source, patient status, underlying disease etc.) that allow useful comparisons between the growing numbers of trials and the underlying methods and characteristics used to generate and describe the drug (in this case DC). Often, this key aspect of drug development (optimizing and describing the purity and potency of the drug) is overlooked. However, one constant regarding the majority of the trials remains; that is the use of CD14^+^ cells as a starting material.

## Monocyte Precursors of DC are Often Altered in Cancer Patients and are Immune Suppressive

**Figure 1:**
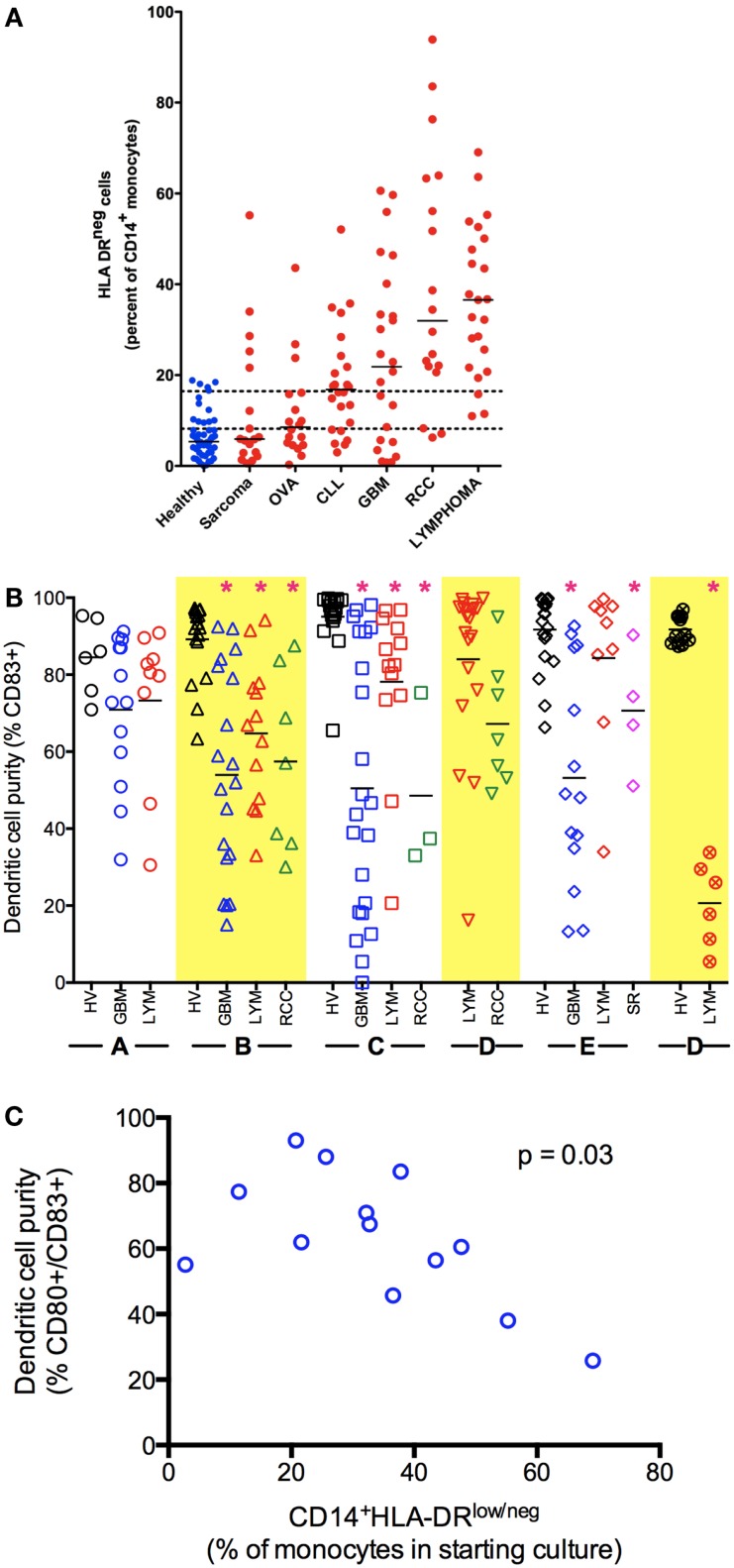
**Monocyte and dendritic cell defects in cancer**. **(A)** Cancer patients have an increased percentage of CD14^+^HLA-DR^lo/neg^ monocytes in circulation. Peripheral blood of healthy volunteers and cancer patients was analyzed by flow cytometry for immune phenotype. **(B)** Monocytes from cancer patients have decreased capacity to differentiate to mDC (CD83^+^) under a variety of stimulation conditions. Monocytes were selected from blood of healthy volunteers (HV) or cancer patients (GBM, glioblastoma multiforme LYM, B-cell lymphoma; RCC, renal cell carcinoma; SR, sarcoma) by CD14^+^ immunomagnetic beads and cultured under different methods as labeled in *X* axis. Method A, fast-DC (28); B, *ex vivo* media with 5 days culture as described (29, 30); C, 5 days culture in StemLine media and GM-CSF, maturation factors TNFα and PGE2 added in the last 2 days of culture; D, method C with IL-4 added for 5 days of culture; E, method D with poly I:C added to maturation factors; F, method D with CpG used as maturation factor in place of TNF-α (*p < 0.05). **(C)** Decreased generation of mDC correlates with increased percentage of CD14^+^HLA-DR^lo/neg^ in the monocytes selected for culture (Method B).

In our efforts to establish a DC vaccine protocol, we worked to optimize the maturation of DCs in cancer patients. During those studies, we discovered that in many patients, CD14^+^-isolated monocytes were incapable of differentiation into mDC using standard DC generation protocols (16–18). This result that monocytes from cancer patients were potentially altered in their capacity to differentiate into DC, led us to search for correlative markers. We identified an increased population of monocytes with an altered surface marker expression (CD14^+^HLA-DR^lo/neg^) in a number of malignancies (16–20) (Figure 1A). This phenotype has also been reported by others in melanoma (21–23), bladder cancer (24), non-small cell lung cancer (25), and hepatocellular cancer (26, 27). Our studies in glioblastoma identified evidence of systemic immune suppression and increased concentrations of CD14^+^HLA-DR^lo/neg^ monocytes capable of inhibiting T-cell proliferation and DC maturation that could also be re-capitulated *in vitro* co-culture systems using tumor cell lines (18). These same immunosuppressive monocytes have been characterized with increased populations in bladder carcinoma that significantly correlate with decreased T-cell proliferation and IFN-γ production (24). These cells suppress immune function in multiple ways (Table 1), and therefore must be considered for any approach to DC vaccine strategies.

**Table 1:** **Methods of CD14^+^HLA-DR^lo/neg^ immune suppression**.

CD14^+^HLA-DR^lo/neg^ functions	Targeted effect	Reference
Altered STAT signals	Resistance to cytokine/TLR signaling	(16, 24)
Increased IDO expression	Inhibits T-cell function	(31–33)
Increased arginase expression	Inhibits T-cell function	(12, 24, 34)
Prevention of DC maturation	Promotes immune tolerance	(16–18)
Altered co-stimulatory expression	Reduces T-cell stimulation	(12, 14, 15, 35)
Altered cytokine expression	Reduces T-cell stimulation	(15, 35)
Decreased antigen uptake	Reduces antigen-specific T-cell responses	(35)
Increased iNOS and NOX2 production	Reduces T-cell stimulation	(36)
Increased VEGF	Inhibits DC differentiation	(37)
Depletion of cytosine	Inhibit T-cell activation	(38, 39)

There is also mounting evidence that correlates increased concentrations of CD14^+^HLA-DR^lo/neg^ monocytes in patients with poor clinical outcome. Populations of CD14^+^HLA-DR^lo/neg^ monocytes and TGF-β levels were significantly expanded in metastatic melanoma patients as compared to healthy donors and correlated to a lack of response to administered granulocyte–macrophage colony-stimulation factor (GM-CSF) vaccine (22). Increased CD14^+^HLA-DR^lo/neg^ monocytes correlated to both extrathoracic metastasis and poor response to chemotherapy in non-small lung cancer patients (25). Increased CD14^+^HLA-DR^lo/neg^ monocytes are associated with more aggressive disease and poorer prognosis in lymphoma (16) and hepatocellular carcinoma (40). Increased CD14^+^HLA-DR^lo/neg^ monocytes were associated with decreased time to progression in patients with chronic lymphocytic leukemia (CLL) (19). Increased CD14^+^HLA-DR^lo/neg^ monocytes and decreased CD4^+^ T cells can predict poor overall survival across a number of malignancies (20).

The finding that CD14^+^HLA-DR^lo/neg^ monocytes are detectable systemically in patients with a variety of malignancies and that they are functionally immune suppressive raises important questions regarding their influence in *ex vivo* DC vaccine preparations. To address this, we have studied the effects of these altered monocytes on mDC generation across several cancer types using an *ex vivo* culture system. Briefly, CD14^+^ mononuclear cells were isolated from buffy coats or apheresis leukoreduction system chambers of normal donors using immunomagnetic selection (41). Control DCs were cultured with 1% human AB serum, stimulated with GM-CSF and IL-4 (base media) for 3 days when one-third volume of fresh base media was added. Non-adherent cells were collected on day 6, re-suspended in base media with the addition of tumor necrosis factor alpha (TNF-α) and prostaglandin E2 (PGE2) to mature the DCs. This recipe is based on a classic method of generation of mDCs (28, 29, 41). Alternatively, the system was modified using a serum-free media and changes in cytokines to generate mDCs (Figure 1B).

Cancer patients showed significant deficits in the ability to generate mDCs independent of the underlying tumor. There is also substantially more variability in the efficiency of DC generation using monocytes from cancer patients (Figure 1B). While we primarily used CD83 up-regulation as indicative of DC maturation, we also noted a lack of CD80 expression and specific functional deficient of these cells. Increased efficiency of DC maturation can be correlated with decreased presence of CD14^+^HLA-DR^lo/neg^ monocytes in the starting culture (Figure 1C). However, we could consistently improve the ability to generate mDCs using serum-free methods with the addition of IL-4. Even so, it was difficult to recapitulate the efficient generation of mDCs we observed using monocytes from healthy volunteers compared to cancer patients. Knowing that CD14^+^HLA-DR^lo/neg^ monocytes have significant capacity to influence *ex vivo* DC cultures implies that these cells and the pathways to both generate and eliminate them are high-value targets to improve cancer therapies. It is striking to note that these effects occurred in the complete absence of tumors and in the continual presence (for days) with the cells in excess cytokines. This deficit likely represents a significant block in the differentiation pathway. *This strongly suggests that immune stimulation in vivo, even with precise targeting of the pathways known to convert mature DC, is unlikely to efficiently trigger mDC maturation in patients*. Further understanding of the biology of these CD14^+^HLA-DR^lo/neg^ monocytes is needed; strategies to overcome the effects of these cells can lead to better DC generation and immune reconstitution.

## Implications of CD14^+^HLA-DR^lo/neg^ on DC-Based Cancer Vaccines

Most currently active DC-based cancer immunotherapy protocols differ in either the cell source or some of the methods associated with the generation of DC. The most common approach has been the *ex vivo* generation of mature DCs from patient myeloid-derived monocyte precursors by co-culturing with GM-CSF and various cocktails of cytokines and TLR agonists to produce mature DCs. Optimizing DC culture conditions using normal healthy donors will likely not directly translate into the protocols needed for cancer patients. It will be important for those protocols that use CD14^+^ cells to generate their DC product from primary patient samples to confirm and optimize the manufacturing method and assure that potent DCs are being generated. In our hands, a serum-free method that includes IL-4 is a good starting point. Adequate sampling size of the patient population is needed to determine the range of differentiation efficiency in each specific cancer patient population to inform the design of release criteria for vaccine manufacturing. Our data also have clear implications for other approaches attempting to mediate anti-tumor immune stimulation. *Adjuvants known to work in healthy people may not work in cancer patients if their approach is to target the DC or DC differentiation pathways*.

As we improve our understanding of the importance of CD14^+^HLA-DR^lo/neg^ monocytes in promoting immunosuppression, it is imperative that we adjust our clinical practices to ensure effective outcomes for patients using DC-based immunotherapy. This will require continued efforts to develop optimal protocols for generating *ex vivo* DC vaccine preparations and testing these protocols in individual patients. The complexity of the human immune system and individual tumor micro environments will likely require an element of individualized protocol development to achieve optimal clinical benefit.

## Conflict of Interest Statement

The authors declare that the research was conducted in the absence of any commercial or financial relationships that could be construed as a potential conflict of interest.
